# A review of *Aleurodaphis* (Hemiptera, Aphididae, Hormaphidinae) with the description of one new species and keys to species

**DOI:** 10.3897/zookeys.135.1721

**Published:** 2011-10-07

**Authors:** Li-Yun Jiang, Ge-Xia Qiao

**Affiliations:** 1 Key Laboratory of Zoological Systematics and Evolution and National Zoological Museum of China, Institute of Zoology, Chinese Academy of Sciences, Beijing 100101, P. R. China

**Keywords:** Hormaphidinae, *Aleurodaphis*, new species, synonym

## Abstract

The genus *Aleurodaphis* van der Goot is reviewed. One new species *Aleurodaphis sinojackiae* Qiao & Jiang, **sp. n.** on *Sinojackia xylocarpa* from Jiangsu and Zhejiang, China is described. *Aleurodaphis sinisalicis* Zhang, 1982 is synonymised with *Aleurodaphis blumeae* van der Goot, 1917. Keys to species, morphological description and features of the new species, host plants, and distribution are provided. The specimens including types are deposited in British Natural History Museum, London (BMNH), Kôgakkan University, Japan and the National Zoological Museum of China, Institute of Zoology, Chinese Academy of Sciences, Beijing, China (NZMC).

## Introduction

*Aleurodaphis* is erected in 1917 by van der Goot. He described the species *Aleurodaphis blumeae* as the type of the genus, from *Blumea*. Its remarkable characters are the followings, body of apterae is aleyrodiform, frontal horn is absent and wax glands are arranged along crenulated margin of body. Takahashi studied the specimens from East Asia, and described two species *Aleurodaphis mikaniae* Takahashi, 1925 and *Aleurodaphis asteris* Takahashi & Sorin, 1958. More than 30 years later, one new species was found from India, *Aleurodaphis antennata* Chakrabarti & Maity, (1980) 1982 and one new species was reported in China, *Aleurodaphis sinisalicis* Zhang, 1982. Sorin and Miyazaki (2004) reviewed the genus from Japan with descriptions of three new species, *Aleurodaphis impatientis*, *Aleurodaphis ligulariae* and *Aleurodaphis stewartiae*. After identifying the specimens from China and checking the specimens of the genus in British Natural History Museum, one new species, *Aleurodaphis sinojackiae* Qiao & Jiang, sp. n. is found, and *Aleurodaphis sinisalicis* Zhang, 1982 is synonymised with *Aleurodaphis blumeae* van der Goot, 1917 here. Therefore, the genus has eight known species in the world (Remaudière and Remaudière 1997; Sorin and Miyazaki 2004), including the new species described here.

## Material and methods

Aphid terminology in this paper follows Sorin and Miyazaki (2004) and Ghosh (1988). The unit of measurements in this paper is millimeters (mm).

In Tables 1-2, the following abbreviations have been used: Ant. I – IV = antennal segments I – IV; Ant. V b = base of antennal segment V; pt = processus terminalis; URS = ultimate rostral segment; Hind T & F = hind trochanter & femur; 2HTs = second hind tarsal segment.

**Table 1.** Measurements of apterous viviparous females of *Aleurodaphis sinojackiae* Qiao & Jiang, sp. n. (mm)

No.	Body length	Body width	Ant. I	Ant. II	Ant. III	Ant. IV	Ant. V b	Ant. V pt	URS	Hind T & F	2HTs	Cauda length
1	1.92	1.15	0.07	0.08	0.21	0.12	0.13	0.04	0.13	0.36	0.10	0.10
2	1.98	1.13	0.07	0.07	0.20	0.10	0.12	0.03	0.15	0.36	0.10	0.09
3*	2.05	1.21	0.07	0.08	0.23	0.12	0.13	0.03	0.14	0.38	0.10	0.09
4	1.89	1.07	0.07	0.08	0.19	0.12	0.12	0.03	0.12	0.33	0.10	0.09
5	1.86	1.09	0.08	0.07	0.21	0.11	0.12	0.04	0.14	0.37	0.11	0.08
6	2.09	1.01	0.08	0.08	0.23	0.12	0.14	0.04	0.13	0.39	0.11	0.09
7	2.05	1.24	0.07	0.08	0.22	0.12	0.13	0.03	0.15	0.37	0.10	0.09
8	2.03	1.05	0.07	0.08	0.22	0.11	0.13	0.04	0.14	0.37	0.10	0.09
Average	1.98	1.12	0.07	0.08	0.21	0.11	0.13	0.03	0.14	0.37	0.10	0.09

Remark. * Holotype; for abbreviations see Materials and Methods.

**Table 2.** Measurements of alate viviparous females of *Aleurodaphis sinojackiae* Qiao & Jiang, sp. n. (mm)

**No.**	**Body length**	**Body width**	**Ant. I**	**Ant. II**	**Ant. III**	**Ant. IV**	**Ant. V b**	**Ant. V pt**	**URS**	**Hind femur**	**2HTs**	**Cauda length**
1	1.84	0.81	0.08	0.07	0.28	0.14	0.14	0.04	0.14	0.40	0.09	0.08
2	2.00	0.82	0.07	0.07	0.25	0.14	0.13	0.04	0.13	0.39	0.11	0.08
4	1.49	0.66	0.06	0.06	0.18	0.11	0.09	0.02	0.10	0.29	0.08	0.06
5	1.45	0.63	0.05	0.05	0.19	0.09	0.08	0.02	0.10	0.30	0.08	0.06
6	1.32	0.64	0.05	0.05	0.21	0.11	0.11	0.03	0.11	0.31	0.08	0.05
7	1.42	0.56	0.06	0.05	0.18	0.11	0.09	0.02	0.11	0.31	0.07	0.06
8	1.30	0.58	0.06	0.05	0.19	0.10	0.09	0.03	0.11	0.26	0.08	0.06
Average	1.45	0.64	0.06	0.05	0.20	0.11	0.10	0.03	0.11	0.30	0.08	0.06

Specimen depositories. The holotype and some paratypes of the new species are deposited in British Natural History Museum, London (BMNH), while the other paratypes in the National Zoological Museum of China, Institute of Zoology, Chinese Academy of Sciences, Beijing, China (NZMC) and Kôgakkan University, Japan. All the other specimens studied are deposited in BMNH and NZMC.

## Systematics

### Aleurodaphis

van der Goot, 1917

http://species-id.net/wiki/Aleurodaphis

Aleurodaphis  van der Goot, 1917: 239.
Aleurodaphis  van der Goot: Baker 1929: 86; Takahashi 1931: 92; Takahashi and Sorin 1958: 31; Raychaudhuri et al. 1980: 36; Ghosh 1988: 249; Noordam 1991: 47; Tao 1990: 58; Blackman and Eastop 1994: 551; Remaudière and Remaudière 1997: 181; Tao 1999: 17.

#### Type species.

 *Aleurodaphis blumae* van der Goot, 1917.

#### Diagnosis.

Body oval and flat. In apterous females: body aleyrodiform, absence of frontal horns, and wax glands arranged along the crenulated margin of body. Head and prothorax, meso- and metathorax, abdominal tergites I–VII fused, respectively; only abdominal tergite VIII free; antennae 4 or 5-segmented, primary rhinaria small and ciliated; eyes with 3 facets. Dorsal setae fine and sparse. Rostrum reaching mid-coxae, at most hind coxae. Ultimate rostral segment obviously longer than second hind tarsal segment. Legs short; first tarsal chaetotaxy: 2–4, 2–4, 2–4; dorsal-apical setae on second hind tarsal segments with funnel-shaped apex. Siphunculi ring-shaped. Cauda knobbed and anal plate bilobed. In alate viviparous females: antennae 5-segmented, with secondary rhinaria near ring-shaped, without cilia; eyes normal; first tarsal chaetotaxy: 4, 4, 4, sometimes 3 or 2; fore wings with media once branched, pterostigma extended and two cubitus fused or separated at base; hind wings with two obliques.

#### Host plants.

The range of host plantsin *Aleurodaphis* is quite wide, including Compositae (*Aster*, *Blumea*, *Carpesium*, *Chrysanthemum*, *Kalimeris*, *Ligularia*, *Parasenecio*, *Senecio*), Balsaminaceae (*Impatiens*), Gramineae (*Bambusa*), Moraceae (*Ficus*), Plantaginaceae (*Plantago*), Scrophulariaceae (*Mazus*), Styracaceae (*Sinojackia*), Theaceae (*Stewartia*), Verbenaceae (*Callicarpa*) and Violaceae.

#### Biology.

Five species, *Aleurodaphis asteris*, *Aleurodaphis blumeae*, *Aleurodaphis impatientis*, *Aleurodaphis ligulariae* and *Aleurodaphis mikaniae*, mainly feeding on Compositae species, have monoecious and anholocyclic life cycle. *Aleurodaphis sinojackiae* Qiao & Jiang, sp. n. and *Aleurodaphis stewartiae* can form galls on the leaves of the primary host plants, but their secondary hosts are unknown. The details of *Aleurodaphis antennata* wereunreported (Ghosh 1988; Blackman and Eastop 1994, 2006; Sorin and Miyazaki 2004).

#### Distribution.

China, Japan, India and Indonesia.

#### Keys to species of Aleurodaphis

Apterous viviparous females

**Table:** 

1	Body without marginal wax glands; on *Stewartia*, in curled leaves	*Aleurodaphis stewartiae*
–	Body with marginal wax glands	2
2	Marginal wax glands arranged in each segment, not connecting with each other (Fig. 20); on *Sinojackia*, in curled leaves	*Aleurodaphis sinojackiae* Qiao & Jiang, sp. n.
–	Marginal wax glands arranged consecutively along the crenulated margin of body (Figs 1, 2), not in curled leaves	3
3	Ultimate rostral segment slender and long, 4.60–5.67 times as long as its basal width (Fig. 2)	*Aleurodaphis blumeae*
–	Ultimate rostral segment stout and short, less than 3.30 times as long as its basal width	4
4	Each first tarsal segment with 2 setae; marginal wax glands along the crenulated margin of body with 120 wax facets at most	*Aleurodaphis asteris*
–	Each first tarsal segment with more than 2 setae; marginal wax glands along the crenulated margin of body with 150 wax facets at least	5
5	Ultimate rostral segment 1.16–1.40 times as long as second hind tarsal segment	6
–	Ultimate rostral segment 1.41–2.02 times as long as second hind tarsal segment	7
6	Ultimate rostral segment 1.16–1.30 times as long as second hind tarsal segment; dorsal of body without obvious mastoid process; on *Bambusa*	*Aleurodaphis antennata*
–	Ultimate rostral segment 1.40 times as long as second hind tarsal segment; dorsal of body with obvious mastoid process; on Compositae and Balsaminaceae (Impatiens)	*Aleurodaphis mikaniae*
7	First tarsal chaetotaxy: 3 or 4, 3 or 4, 3 or 4; triommatidia elongate, with the outer-most facet placed widely apart from the other two; on *Impatiens*	*Aleurodaphis impatientis*
–	First tarsal chaetotaxy: 2 or 3, 2 or 3, 2; triommatidia thickset, with the outer-most facet placed close to the other two; on *Ligularia*	*Aleurodaphis ligulariae*

Alate viviparous females

**Table:** 

1	Antennae 4-segmented, with more than 30 setae on each of segments III and IV	*Aleurodaphis ligulariae*
–	Antennae 5-segmented, with 2–5 setae on each of segments III and IV	2
2	Ultimate rostral segment less than 3.00 times as long as its basal width	3
–	Ultimate rostral segment more than 3.00 times as long as its basal width	4
3	Antennal segment III with 10–14 secondary rhinaria; first tarsal chaetotaxy: 4, 4, 4, sometimes 3, 3, 3	*Aleurodaphis sinojackiae* Qiao & Jiang, sp. n.
–	Antennal segment III with 24–27 secondary rhinaria; first tarsal chaetotaxy: 3, 3, 3, sometimes 2, 2, 2	*Aleurodaphis mikaniae*
4	Ultimate rostral segment 4.60–7.50 times as long as its basal width, and 1.96–2.32 times as long as second hind tarsal segment	*Aleurodaphis blumeae*
–	Ultimate rostral segment 3.38–4.00 times as long as its basal width, and 1.19–1.80 times as long as second hind tarsal segment	5
5	First tarsal chaetotaxy: 3, 3, 3; antennal segment III with 25–29 secondary rhinaria	*Aleurodaphis stewartiae*
–	First tarsal chaetotaxy: 4, 4, 4 or 3–4, 3–4, 2–4; antennal segment III with 9–12 secondary rhinaria	*Aleurodaphis impatientis*

### Aleurodaphis antennata

Chakrabarti & Maity, (1980) 1982

http://species-id.net/wiki/Aleurodaphis_antennata

Aleurodaphis antennata  Chakrabarti & Maity, (1980) 1982: 56.
Aleurodaphis antennata  Chakrabarti & Maity: Ghosh 1988: 252; Blackman and Eastop 1994: 551; Remaudière and Remaudière 1997: 179.

#### Host plants.

*Bambusa* sp.

#### Distribution.

India (Ghosh 1988) .

### Aleurodaphis asteris

Takahashi & Sorin, 1958

http://species-id.net/wiki/Aleurodaphis_asteris

Aleurodaphis asteris  Takahashi & Sorin, 1958: 31.
Aleurodaphis asteris  Takahashi & Sorin: Zhang et al. 1992: 142; Remaudière and Remaudière 1997: 179; Tao 1999: 17; Sorin and Miyazaki 2004: 166.

#### Material examined.

 **CHINA** (NZMC): 2 apterous viviparous females, 15 April 1991, Jiangle, Fujiang, No. 10054, on Violaceae, coll. W. Y. Zhang; 8 apterous viviparous females, 13 August 2003, Motuo, Tibet, No. 15371, host plants unknown, coll. G. X. Qiao and X. L. Huang; **JAPAN** (BMNH):24 apterous viviparous females, 5 August 1966, Osaka, Chihaya, on *Aster* sp., coll. M. Sorin.; 9 apterous viviparous females, 29 May 1964, Osaka, Kongo Mt., on *Aster* sp., coll. v. d. Bosch; 4 apterous viviparous females, 7 June 1966, Kyushu, Hikosan, on *Kalimeris* sp., coll. H. Takada; 8 apterous viviparous females, 6 August 1980, Kyoto, Kibune Mt., on *Aster yomena*, coll. R. L. Blackman; **KOREA** (BMNH):2 apterous viviparous females, 15 September 1963, Ulnungdo, on *Aster incisus*, coll. W. H. Paik; 1 apterous viviparous female, 14 September 1963, Pusan, on *Chrysanthemum zawidskii*, coll. W. H. Paik.

#### Host plants.

*Carpesium abrotanoides*, *Aster yomena*, *Aster incisus*, *Chrysanthemum zawaidskii*, *Kalimeris* sp. and Violaceae.

#### Biology.

The species feed on the stems, leafstalks, flower stalks and leaves of the host plants.

#### Distribution.

China, Japan and Korea.

### Aleurodaphis blumeae

van der Goot, 1917

http://species-id.net/wiki/Aleurodaphis_blumeae

Figs 1–2

Aleurodaphis blumeae  van der Goot, 1917: 240.
Aleurodaphis nobukii  Shinji, 1923: 301.
Astegopteryx japonica  Takahashi, 1923: 150.
Aleurodaphis sinisalicis  Zhang, 1982: 20. syn. n.
Aleurodaphis blumeae  van der Goot: Takahashi 1921: 92; Takahashi 1923: 150; Takahashi 1924: 98; Raychaudhuri et al. 1980: 362; Ghosh 1988: 256; Noordam 1991: 47; Tao 1990: 59; 1999: 17; Remaudière and Remaudière 1997: 179; Sorin and Miyazaki 2004: 166.

#### Comments.

**Figures 1–2.**
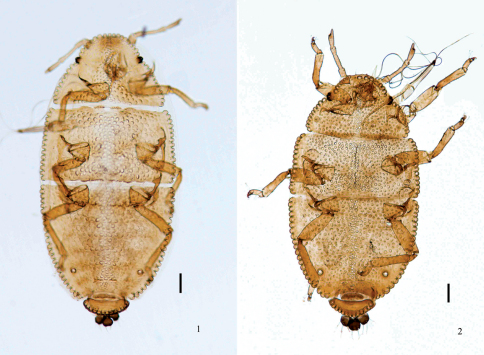
Apterous viviparous female. **1** dorsal view of body, *Aleurodaphis blumeae* van der Goot **2 **dorsal view of body, syntype of *Aleurodaphis sinisalicis* Zhang. Scale bars = 0.10 mm.

The type specimens of *Aleurodaphis sinisalicis* Zhang, 1982 were checked, including 48 apterous viviparous females, 25 July 1963, Sichuan (Guanxian County), No. Y0399, on *Salix* sp., coll. G. X. Zhang and T. S. Zhong. The result confirmed the queries of Blackman and Eastop (1994) and Remaudière and Remaudière (1997) that *Aleurodaphis sinisalicis* (Fig. 2) was a synonym of *Aleurodaphis blumeae* (Fig. 1).

The original descriptions of *Aleurodaphis sinisalicis* Zhang, 1982 were accurate, but the morphological characters of *Aleurodaphis blumeae* in his diagnosis were wrong. Perhaps, this is the main reason why Zhang (1992) described it as a new species. In the original descriptions of Aleurodaphis sinisalicis Zhang, 1982, the diagnosis was: the ratio of body length to antennae length was 4.70 (*Aleurodaphis blumeae*: 2.70), the base of cauda restricted (*Aleurodaphis blumeae*: not restricted), and the anal plate bilobed (*Aleurodaphis blumeae*: not bilobed). Actually, the morphological characters of *Aleurodaphis blumeae* in this diagnosis were inaccurate. In A. blumeae, the ratio of body length to antennae length was 4.80 instead of 2.70, the base of cauda restricted instead of not restricted, and the anal plate bilobed instead of not bilobed.

The host plant of *Aleurodaphis sinisalicis*, *Salix* sp., is perhaps mis-recorded.

#### Material examined.

 **CHINA** (NZMC): 6 apterous viviparous females, 17 August 2004, Guizhou (Daozhen County), No. 15597, host plants unknown, coll. J. Y. Yang; 6 apterous viviparous females, 17 July 2001, Shaanxi (Nanzheng County), No. Y8606, host plants unknown, coll. S. H. Wang; 7 apterous viviparous females, 8 September 1995, Jiangxi (Jinggangshan City), No. 10852, on Compositae, coll. G. X. Zhang; 9 apterous viviparous females and 7 alate viviparous females, 25 April 1984, Shaanxi (Yangling County), No. 64, on *Carpesium cernuum*, coll. X. F. Dai; 5 alate viviparous females, May 1984, Shaanxi (Yangling County), No. Y6227, host plants unknown, coll. X. F. Dai; 3 apterous viviparous females, 26 June 1983, Zhejiang (Lin’an City), No. Y2692, on *Carpesium abrotanoides*, collector unknown; 5 apterous viviparous females, 8 April 1998, Guangxi (Napo County), No. 11772, on *Callicarpa bodinieri*, coll. G. X. Qiao; 14 apterous viviparous females, 21 April 1998, Guangxi (Fangchenggang City), No. 11840, on Senecio *scandens*, coll. G. X. Qiao; 16 apterous viviparous females, 22 March 1998, Guangxi (Pingxiang City), No. 11580, on *Plantago asiatica*, coll. G. X. Qiao; 7 apterous viviparous females, Hunan, No. 8887, on Compositae, the collector unknown; 3 apterous viviparous females and 2 nymphs, Feburary 1925, Taiwan (Taihoku), on *Ficus* sp., coll. R. Takahashi (BMNH); 107 apterous viviparous females, 1 alate viviparous female and 24 nymphs, 21 May 1985, Zhejiang (Hangzhou City), on *Carpesium abrotanoides*, coll. V. F. Eastop (BMNH); **JAPAN** (BMNH):2 apterous viviparous females and 4 nymphs, 29 August 1913, Kumamoto, on *Blumea* sp., coll. Theobald; 10 alate viviparous females, 22 September 1957, Osaka, on *Carpesium abrotanoides var. tumbergianum*, coll. M. Sorin; 2 apterous viviparous females, 2 alate viviparous females and 4 nymphs, 30 July 1957, Tokyo, Takao Mt., on *Blumea* sp., coll. R. Takahashi; 7 apterous viviparous females and 12 nymphs, 16 August 1991, Chiba, Sayama, on *Carpesium* sp., coll. D. L. Stern; **KOREA** (BMNH): 2 apterous viviparous females, 15 September 1963, Ulnungdo, on *Mazus miguelii*, coll. W. H. Paik; 1 apterous viviparous female, July 1969, Lri, host plants unknown, coll. W. H. Paik; **INDONESIA** (BMNH): 6 apterous viviparous females and 2 alate viviparous females, 13 July 1916, Garoet, on Compositae, coll. D. van der Goot; **MALAYSIA** (BMNH): 3 apterous viviparous females and 2 nymphs, 23 September 1944,Cameron Highlands, on *Blumea* sp., coll. R. Takahashi; **PHILIPPINES** (BMNH):1 alate viviparous female, September 1962, Davao Exp. Station, trap in Abacca grove, coll. M. R. Gavarra; 1 alate viviparous female, July 1963, Davao Exp. Station, host plants unknown, coll. M. R. Gavarra; 1 alate viviparous female, January 1964, Davao Exp. Station, host plants unknown, coll. M. R. Gavarra; 1 alate viviparous female, March 1964, Davao Exp. Station, host plants unknown, coll. M. R. Gavarra; 3 apterous viviparous females and 2 nymphs, 13 September 1964, Makiling, on *Blumea* sp., coll. V. S. Calilung.

#### Host plants.

*Carpesium cernuum*, *Carpesium abrotanoides*, *Carpesium abrotanoides* var. *tumbergianum*, *Senecio scandens*, *Blumea chinensis*, *Callicarpa bodinieri*, *Mazus miguelii*, *Ficus* sp. and *Plantago asiatica*. The common hosts are various Compositae.

#### Biology.

This species feeds on the lower surface of leaves, along the main veins. It can infest *Blumea* on stems and undersides of young leaves, causing slight leaf-curl (Calilung 1967).

#### Distribution.

China, Japan, Korea, Indonesia, Malaysia and Philippine.

### Aleurodaphis impatientis

Sorin & Miyazaki, 2004

http://species-id.net/wiki/Aleurodaphis_impatientis

Aleurodaphis impatientis  Sorin & Miyazaki, 2004: 167.

#### Host plants.

*Impatiens textori* and *Impatiens noli-tangere*.

#### Biology.

The species is viviparous throughout the year on *Impatiens* spp. Alate viviparous females appear in the latter part of September. Adult apterous viviparous females pass the winter on the stalks near and just below the ground level. The hibernated adult females move to the seedlings and start to feed on the leaves and stalks in mid-April (Sorin and Miyazaki 2004).

#### Distribution.

Japan (Sorin and Miyazaki 2004).

### Aleurodaphis ligulariae

Sorin & Miyazaki, 2004

http://species-id.net/wiki/Aleurodaphis_ligulariae

Aleurodaphis ligulariae  Sorin & Miyazaki, 2004: 170.

#### Host plant.

*Ligularia fischeri*.

#### Biology.

The aphid lives on the lower side of the leaves and the apical part of the stem, as well as on the flower stalk at the tips of the host plant shoots. The alate viviparous females appear in the latter part of September. Adult apterous viviparous females pass the winter on the basal part of the stem just below the ground level and on fallen leaves in the ground litter (Sorin and Miyazaki 2004).

#### Distribution.

Japan (Sorin and Miyazaki 2004).

### Aleurodaphis mikaniae

Takahashi, 1925

http://species-id.net/wiki/Aleurodaphis_mikaniae

Aleurodaphis mikaniae  Takahashi, 1925: 51.
Aleurodaphis mikaniae  Takahashi: Remaudière and Remaudière 1997: 179; Tao 1999: 17.

#### Material examined.

 **CHINA** (NZMC): 6 apterous viviparous females and 3 alate viviparous females, 24 August 2004, Guizhou (Daozhen County), No. 15638, host plants unknown, coll. J. Y. Yang; 4 apterous viviparous females, 15 August 2003, Sichuan (Baoxing County), No. 15017, on *Parasenecio* sp., coll. K. Guo; 7 apterous viviparous females, 27 June 1999, Shaanxi (Foping County), No. 12336, on Compositae, coll. T. L. He; 20 apterous viviparous females, 12 October 1988, Hunan (Zhangjiajie City), No. 8962, on *Impatiens* sp., coll. T. S. Zhong and G. X. Zhang; 4 apterous viviparous females, 26 September 1974, Guizhou (Guiyang City), No. Y2123, on *Senecio scandens*, coll. Y. Y. Rao; 10 apterous viviparous females, 31 March 1982, Yunnan (Kunming City), No. 7373, on *Senecio scandens*, coll. G. X. Zhang; 6 apterous viviparous females, 12 October 1996, Shaanxi (Zhouzhi County), No. 11096, host plants unknown, coll. G. X. Qiao; 4 apterous viviparous females, 18 August 2003, Sichuan (Baoxing County), host plants unknown, coll. K. Guo; 10 apterous viviparous females, 12 July 2002, Shaanxi (Meixian County), No. 13559, host plants unknown, coll. E. B. Ma; 14 apterous viviparous females, July 1936, Taiwan (Shinkwan), host plants unknown, coll. R. Takahashi; **JAPAN** (BMNH): 41 apterous viviparous females, 6 August 1980, Kyoto, Kibune Mt., on *Impatiens* sp., coll. R. L. Blackman.

#### Host plants.

*Parasenecio* sp., *Impatiens* sp., and *Senecio scandens*.

#### Distribution.

China and Japan.

### Aleurodaphis sinojackiae

Qiao & Jiang sp. n.

urn:lsid:zoobank.org:act:4FE949A7-BF92-425C-BEDC-176EB4CA495A

http://species-id.net/wiki/Aleurodaphis_sinojackiae

Figs 3 [Fig F3] 34 

#### Locus typicus.

China (Jiangsu and Zhejiang).

#### Etymology.

The new species is named after its host plant, *Sinojackia xylocarpa*.

#### Description of mounted specimens.

*Apterous viviparous* females (Table 1; Figs 3–13, 20–28). Body oval (Fig. 20). Measurements: body 1.86–2.09 long, 1.01–1.24 wide. Cephalic setae, marginal setae on abdominal tergite I and dorsal setae on abdominal tergite VIII 0.04–0.06, 0.04, 0.06–0.07 long. Antennae 0.61–0.68 long, segment III 0.19–0.23 long. Setae on segment III 0.03 long. Ultimate rostral segment 0.12–0.15 long. Hind trochanter and femur 0.33–0.39 long, hind tibia 0.38–0.44 long, second hind tarsal segment 0.10–0.11 long. Setae on hind tibia 0.04–0.06 long. Apical diameter of siphunculi 0.04–0.05. Cauda 0.08–0.10 long.

**Figures 3–19.**
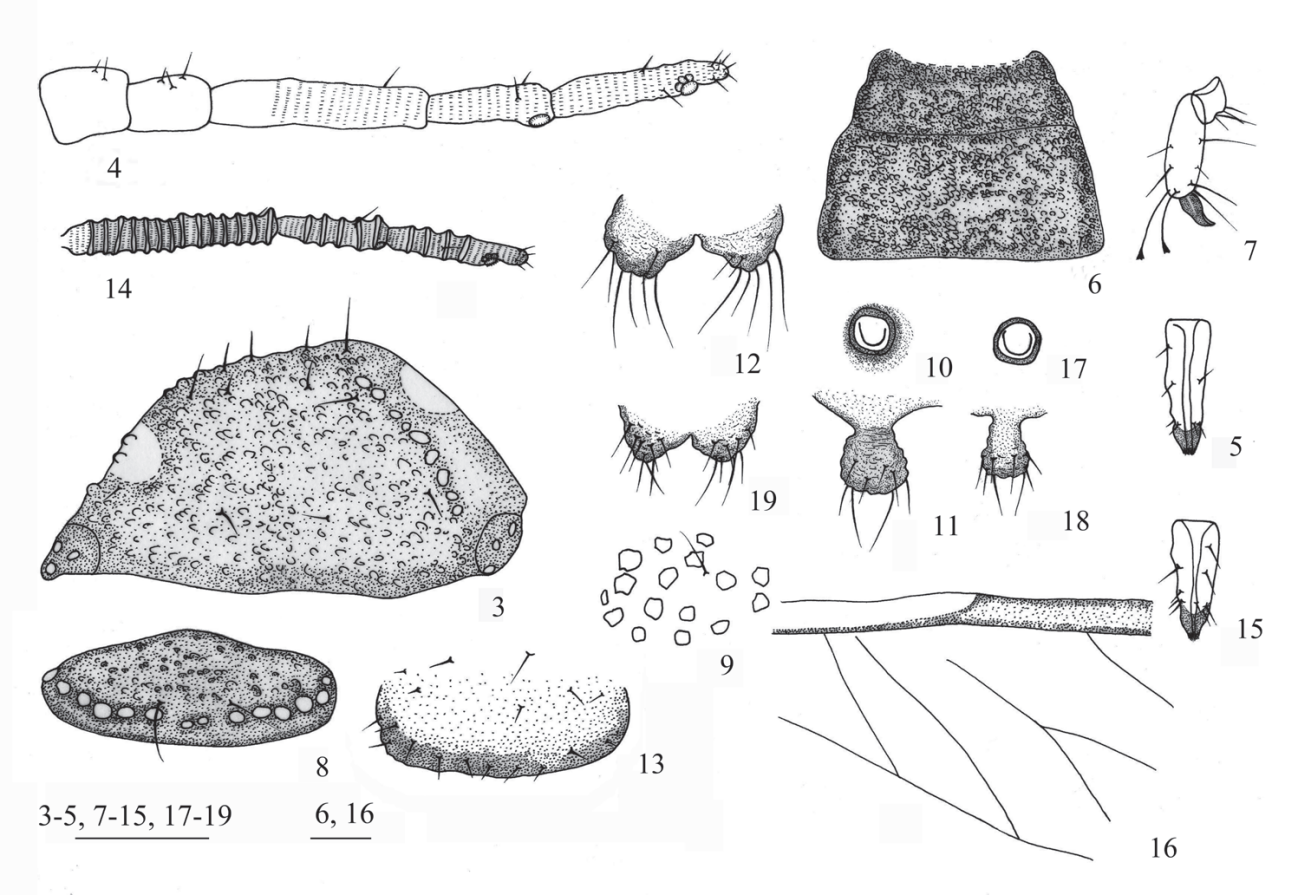
*Aleurodaphis sinojackiae* Qiao & Jiang, sp. n. **3–13** Apterous viviparous female. **3** dorsal view of head **4** antennae **5** ultimate rostral segment **6** dorsal view of thorax **7** hind tarsal segments **8** abdominal tergite VIII **9** dorsal setae and sculptures on abdominal tergite VI **10** siphunculus **11** cauda **12** anal plate **13** genital plate. **14–19** Alate viviparous female. **14** antennae 15 ultimate rostral segment **16** basal half of fore wing **17** siphunculus **18** cauda **19** anal plate. Scale bars = 0.10 mm.

**Figures 20–28. F3:**
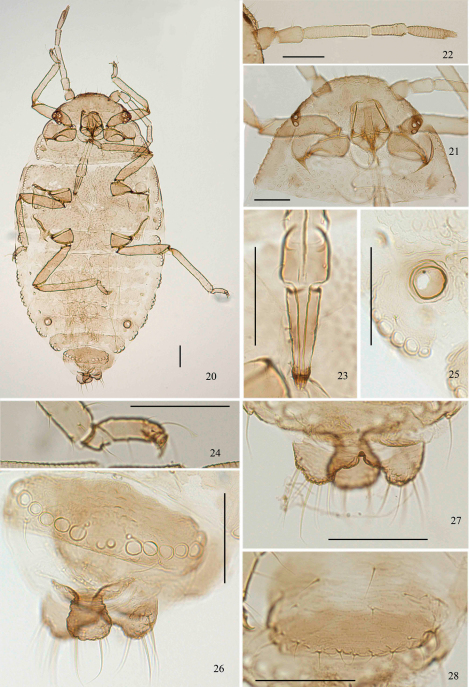
*Aleurodaphis sinojackiae* Qiao & Jiang, sp. n. Apterous viviparous female. **20** dorsal view of body **21** dorsal view of head and pronotum **22** antenna **23** ultimate rostral segment **24** fore tarsal segments **25** siphunculus **26** abdominal tergite VIII and cauda **27** anal plate **28** genital plate. Scale bars = 0.10 mm.

Head and pronotum (Figs 3, 21), mesonotum and metanotum (Fig. 6), abdominal tergites I–VII fused (Fig. 20), respectively; tergite VIII free (Fig. 8). Antennae, rostrum and legs brown; cauda, anal plate and genital plate dark brown. Dorsum of body rough, covered with dense sculptures on dorsum of head and thoracic notums, and with sparse sculptures on abdominal tergites (Fig. 9). Dorsum of body with round marginal wax glands, composited with big facets (Figs 8, 25, 26). Pro- and metanotum each with 13 wax glands, mesonotum with 8 wax glands, abdominal tergites I–VII each with 3–6 pairs of wax glands; tergite VIII with 10–13 wax glands. Dorsal setae of body fine and short (Fig. 9). Head with 2 pairs of cephalic and spinal setae, 3 pairs of marginal setae; pronotum with 2 pairs of spinal, 1 pair of pleural and 2 pairs of marginal setae; mesonotum with 2 pairs of spinal, 3 pairs of pleural and 2 pairs of marginal setae; metanotum with 1 pair of spinal, 3 pairs of pleural and 2 pairs of marginal setae; abdominal tergite I with 1 pair of spinal, pleural and marginal setae; tergites II–VII each with 1 pair of spinal and marginal setae; tergite VIII with 1 pair of spinal and 5 marginal setae. Cephalic setae, marginal setae on abdominal tergite I, setae on abdominal tergite VIII 1.20–1.60, 0.45–1.20 and 1.09–2.00 times as long as widest diameter of antennal segment III, respectively. Spiracles oval, closed, on brown oval spiracular plates.

Head: Front flat and straight. Eyes with 3 facets. Antennae 5-segmented (Figs 4, 22), with spinulose imbrications on segments III–V, 0.33–0.38 times as long as body. Length in proportion of segments I–V: 31–38 : 34–42 : 100 : 50–62 : 56–65+13–21, respectively. Processus terminalis 0.25–0.33 times as long as base of the segment V. Segments I–V each with 2–4, 2 or 3, 0 or 1, 1 or 2, 2+0 setae, respectively. Processus terminalis with 5 or 6 apical setae. Setae on segment III 0.58 times as long as widest diameter of the segment. Primary rhinaria small and round. Rostrum short, reaching mid-coxae. Ultimate rostral segment acute wedge-shaped (Figs 5, 23), 2.67–3.33 times as long as its basal width, 1.14–1.43 times as long as second hind tarsal segment; with 2 pairs of primary setae and 1 or 2 pairs of secondary setae.

Thorax: Mesosternal furca with two separated arms, each arm 1.21–1.41 times as long as widest diameter of antennal segment III. Legs normal. Trochanter and femora fused, hind trochanter and femur 1.63–1.85 times as long as antennal segment III, hind tibia 0.20–0.22 times as long as body; setae on hind tibia 0.88–0.94 times as long as its mid-diameter. First tarsal chaetotaxy: 4, 4, 4, sometimes 3, 3, 4 or 4, 4, 3. Second hind tarsal segment with 2 setae between claws and each seta with funnel-shaped apex (Figs 7, 24).

Abdomen: Siphunculi pore-like (Figs 10, 25), on abdominal tergite VI, apical diameter 1.00–1.40 times as long as widest diameter of antennal segment III. Cauda, anal plate and genital plate with spinulose imbrications. Cauda knobbed (Figs 11, 26), constricted in middle, 0.55–0.68 times as long as its basal width, with 9 or 10 apical setae. Anal plate bilobed (Figs 12, 27), each with 6–8 setae. Genital plate broad band-shaped (Figs 13, 28), with 3 or 4 anterior setae and 14–23 middle and posterior marginal setae. Two gonapophyses each with 5 short setae.

*Alate viviparous* females (Table 2; Figs 4–19, 29–34). Body oval (Fig. 29). Measurements: body 1.30–2.00 long, 0.56–0.82 wide. Cephalic setae, marginal setae on abdominal tergite I and dorsal setae on abdominal tergite VIII 0.016–0.021, 0.015–0.017, 0.020–0.026 long, respectively. Antennae 0.49–0.74 long, segment III 0.18–0.28 long. Ultimate rostral segment 0.10–0.14 long. Hind femur 0.26–0.40 long, hind tibia 0.36 long, second hind tarsal segment 0.07–0.11 long. Setae on hind tibia 0.030 long. Fore wing 1.64–1.74 long. Apical diameter of siphunculi 0.04–0.05. Cauda 0.05–0.08 long.

Dorsum of body dark brown, antenna, apex of rostrum, legs, cauda, anal plate and genital plate brown. Dorsal setae of body fine, short and pointed, slightly longer than ventral setae. Head with 2 pairs of cephalic setae, 2 pairs of setae between antennae and 2 pairs of setae between eyes; abdominal tergites I–VII each with 1 pair of spinal and marginal setae; tergite VIII with 1 pair of spinal setae. Cephalic setae, marginal setae on abdominal tergite I, setae on abdominal tergite VIII 0.51–0.67, 0.50–0.54 and 0.64–0.83 times as long as widest diameter of antennal segment III, respectively.

Head: Front rounded. Antennae 5-segmented (Figs 14, 30), with sparse imbrications on segments I–II and dense spinulose imbrications on segments III–V. Whole length of antennae 0.37–0.43 times as long as body, length in proportion of segments I–V: 21–32 : 24–33 : 100 : 49–60 : 41–53+10–15, respectively. Processus terminalis 0.29–0.37 times as long as base of the segment V. Segments I–V each with 3–5, 2 or 3, 0 or 1, 1, 1 or 2+0 setae, respectively. Processus terminalis with 5 apical setae. Primary rhinaria irregular ring-shaped. Segments III, IV and base of Segment V each with 10–14, 3–6 and 2–4 secondary rhinaria, respectively. Rostrum short, reaching mid-coxae. Ultimate rostral segment (Figs 15, 31) 2.50–2.86 times as long as its basal width, 1.20–1.54 times as long as second hind tarsal segment; with 2 or 3 pairs of primary setae and 1 or 2 pairs of secondary setae.

**Figures 29–34.**
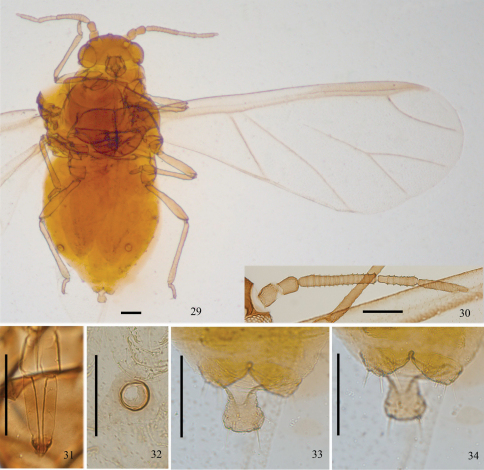
*Aleurodaphis sinojackiae* Qiao & Jiang, sp. n. Alate viviparous female. **29** dorsal view of body **30** antennae **31** ultimate rostral segment **32** siphunculus **33** cauda **34** anal plate. Scale bars = 0.10 mm.

Thorax: Legs normal. Hind femur 1.50–1.62 times as long as antennal segment III, hind tibia 0.25–0.28 times as long as body; setae on hind tibia 0.91–1.20 times as long as its mid-diameter. First tarsal chaetotaxy: 4, 4, 3. Fore wing (Figs 16, 29) 1.17–1.34 times as long as body, 2.00–2.42 times as long as width of the wing. Media once branched. Pterostigma long and curved to the apex of the wing. Hind wings with one thick longitudinal vein and two oblique veins.

Abdomen: Siphunculi pore-like (Figs 17, 32), apical diameter 1.33–1.51 times as long as widest diameter of antennal segment III. Cauda knobbed (Figs 18, 33), constricted in middle, 0.68–0.86 times as long as its basal width, with 6–8 apical setae. Anal plate bilobed (Figs 19, 34), each with 6 or 7 setae. Genital plate broad band-shaped, with 3 anterior setae and 12–15 posterior marginal setae. Two gonapophyses each with 5 or 6 setae.

*Embroys*. Body oval, with wax glands arranged along crenulated margin in both apterae and alatae. Cephalic setae short and pointed. Antennae 4-segmented. Rostrum and legs well developed. Legs covered with dense setae. Siphunculi visible.

#### Type material examined.

Holotype, 1 apterous viviparous female, **CHINA:** Zhejiang (Hangzhou City), 21 May 1985, on *Sinojackia xylocarpa*, coll. V. F. Eastop (BMNH). Paratypes, 28 apterous viviparous females, 2 alate viviparous females and 8 nymphs, with the same collection data as holotype (BMNH); 9 apterous viviparous females and 11 alate viviparous females, **CHINA:** Jiangsu (Zhongshan Botanic Garden, Nanjing City, Alt. 100m), No. Y7116, 10 June 1987, on *Sinojackia xylocarpa*, coll. T. S. Zhong (NZMC); 1 apterous viviparous female and 1 alate viviparous female, **CHINA:** Jiangsu (Zhongshan Botanic Garden, Nanjing City, Alt. 100m), No. Y7116, 10 June 1987, on *Sinojackia xylocarpa*, coll. T. S. Zhong (Kôgakkan University, Japan).

#### Host plants.

*Sinojackia xylocarpa*.

#### Biology.

The species induced the leaves of host plants to curl and form boat-shaped leaf galls.

#### Diagnosis.

The new species differs from the other known speciesas follows: in apterous viviparous female: wax glands arranged in each segment, not connecting with each other (the other species: arranged continuously along the edge of body as a crenulation, or without wax glands); in alate viviparous female compared to the most similar species *Aleurodaphis mikaniae*: antennal segment III with 10–14 secondary rhinaria (*Aleurodaphis mikaniae*: 24–27); first tarsal chaetotaxy: 4, 4, 4, sometimes 3, 3, 3 (*Aleurodaphis mikaniae*: 3, 3, 3, sometimes 2, 2, 2).

#### Remark.

 As the detailed biological information is very important to research the taxonomic position of the genus and species identification, the life cycle of the new species will receive further study in future.

### Aleurodaphis stewartiae

Sorin & Miyazaki, 2004

http://species-id.net/wiki/Aleurodaphis_stewartiae

Aleurodaphis stewartiae  Sorin & Miyazaki, 2004: 174.

#### Host plants.

Primary host: *Stewartia monadelpha*. Secondary hosts unknown.

#### Biology.

The aphid induces a leaf gall, which is formed by rolling the marginal part of the leaf upwards. The gall is about 47.5 long and 7.2 wide, with a surface rough to the touch. The alate viviparous females emerge in early August, and then disappear from the host tree, probably emigrating to some unknown secondary host (Sorin and Miyazaki 2004).

#### Distribution.

Japan (Sorin and Miyazaki 2004).

## Supplementary Material

XML Treatment for Aleurodaphis

XML Treatment for Aleurodaphis antennata

XML Treatment for Aleurodaphis asteris

XML Treatment for Aleurodaphis blumeae

XML Treatment for Aleurodaphis impatientis

XML Treatment for Aleurodaphis ligulariae

XML Treatment for Aleurodaphis mikaniae

XML Treatment for Aleurodaphis sinojackiae

XML Treatment for Aleurodaphis stewartiae
